# MiR-486 regulates cardiomyocyte apoptosis by p53-mediated BCL-2 associated mitochondrial apoptotic pathway

**DOI:** 10.1186/s12872-017-0549-7

**Published:** 2017-05-10

**Authors:** Yuhan Sun, Qiang Su, Lang Li, Xiantao Wang, Yuanxi Lu, Jiabao Liang

**Affiliations:** Department of Cardiology, The First Affiliated Hospital of Guangxi Medical University, Nanning, 530021 China

**Keywords:** Cardiomyocyte, Apoptosis, miR-486, P53, BCL-2 pathway

## Abstract

**Background:**

Cardiomyocyte apoptosis is a common pathological manifestation that occurs in several heart diseases. This study aimed to explore the mechanism of microRNA-486 (miR-486) in cardiomyocyte apoptosis by interfering with the p53-activated BCL-2 associated mitochondrial pathway.

**Methods:**

miR-486 mimics and inhibitors were transfected into the primary cardiomyocytes of suckling Sprague-Dawley rat pups, and H_2_O_2_ was used to induce apoptosis. Flow cytometry and TUNEL were both used to detect cardiomyocyte apoptosis, while the relative mRNA transcript and protein levels of miR-486, p53, Bbc3, BCL-2, and cleaved caspase-3 were detected using RT-PCR and western blot analysis, respectively.

**Results:**

miR-486 overexpression significantly decreased the expressions of p53, Bbc3 and cleaved caspase-3 (*P* < 0.05), and BCL-2 expression was significantly increased (*P* < 0.05), which in turn caused a significant decrease in the rate of cardiomyocyte apoptosis (*P* < 0.05). In contrast, miR-486 silencing resulted in an elevated rate of cardiomyocyte apoptosis (*P* < 0.05).

**Conclusion:**

miR-486 may regulate cardiomyocyte apoptosis via p53-mediated BCL-2 associated mitochondrial apoptotic pathway. Therefore, up-regulating miR-486 expression in cardiomyocytes can effectively reduce the activation of the BCL-2 associated mitochondrial apoptotic pathway, consequently protecting cardiomyocytes.

## Background

Cardiomyocyte apoptosis is a common pathological manifestation in ischemic heart disease, cardiomyopathy, heart failure, and other heart diseases. It can lead to varying degrees of decline in cardiac systolic function, decreased pump function, disorder of electrical activities, severe heart failure, and even sometimes death [1–3]. Cardiomyocyte apoptosis has diverse functions and complex activation mechanisms, and it is the programmed outcome of various pathways ultimately leading to death of the cardiomyocyte [4–6]. We previously reported that cardiomyocytes undergo a range of apoptotic responses under different pathological conditions. During apoptosis of cardiomyocytes, the expression levels of apoptosis-associated caspase family and BCL-2 family proteins are significantly altered, and the mitochondrial and death receptor pathways are significantly activated [7, 8].

P53 is an important apoptosis-associated gene that regulates the apoptotic factor via the Bbc3 effector, death signal receptor pathway, Bax/BCL2, NF receptor, Fas protein, and via other pathways [9]. Yu et al. found that under oxidative stress stimulation, apoptosis of cardiomyocytes and p53 up-regulation occurred together [10]. Raut et al. found that the p53-p21 signaling pathway was activated and was positively correlated with apoptosis under high-glucose induced cardiomyocyte apoptosis [11]. Our previous experiments also revealed that a significant number of cardiomyocytes underwent apoptosis during coronary microembolism and in the inflammatory environment, which was accompanied by significant activation of the p53-Bbc3 mediated mitochondrial apoptotic pathway. However, the mechanism by which p53 activation is regulated, and its involvement in apoptosis of cardiomyocytes remain unknown.

MicroRNAs (miR, miRNAs) serve as a negative regulator of gene expression by promoting mRNA hydrolysis or by inhibiting its translation [12]. miRNAs are also involved in apoptosis [13, 14], in particular, miRNAs have been showed to regulate cardiomyocyte apoptosis, which is involved in the pathogenesis of heart diseases such as myocardial infarction and heart failure. Consequently, regulating the expression of these miRNAs can be a possible treatment approach for these diseases [15, 16]. Nevertheless, it remains unclear whether miRNA can trigger or inhibit P53-Bbc3 mediated mitochondrial apoptotic pathway in cardiomyocytes. In our preliminary experiment, we used microarray analysis to reveal that miR-486 significantly down-regulated cardiomyocyte apoptosis and was negatively correlated with p53 and cardiomyocyte apoptosis. Further research is required to ascertain whether miRNA can regulate p53 and its downstream apoptotic pathway.

Thus, this study aimed to observe the expressions of miR-486 and P53 in apoptotic cardiomyocytes in vitro as well as their dynamic relationship with BCL-2 pathway. The role of miR-486 expression level in the regulation of P53-mediated BCL-2 associated mitochondrial apoptotic pathway in cardiomyocytes was also investigated.

## Methods

### Animals

This experiment was approved by the Ethics Committee of Guangxi Medical University, China and was carried out in accordance with the Norms on the Animal Experiments. The animals were sampled and managed in accordance with guidelines of the National Institute of Health (NIH Publication NO. 85-23, revised 1996).

### In vitro culture and purity testing

Ventricular cardiomyocytes were collected from neonatal Sprague-Dawley rats (1-3 days of age), and then chopped and digested several times in 0.04% collagenase II at 37 °C. The supernatant was then collected, to which 10% fetal bovine serum (FBS) was added to stop the digestion. The supernatant was then filtered with a cell sieve, centrifuged and resuspended in Dulbecco’s modified Eagle’s medium (DMEM)/F-12 (Hyclone, Beijing, China) containing 10% FBS (Gibco, Australia) and 1% mycillin (Solarbio, Beijing, China), inoculated in a culture flask for 1.5 h at 37 °C and 5% CO_2_ to remove the fibroblasts by differential adhesion. The separated cell suspension was seeded in wells of a 24-well culture plates (5 × 10^4^cells/cm^2^), and treated with 0.1 mmol/L 5-bromodeoxyuridine. The medium was replaced every 24 h. Before the follow-up experiments, cardiac troponin I (cTnI) was used to detect the purity of cardiomyocytes using immunofluorescence. Briefly, the cardiomyocytes were stained with anti-cardiac troponin I (Abcam, Cambridge, USA) and fluorescein isothiocyanate (FITC)-conjugated anti-polyclonal IgG (Abcam, Cambridge, USA). For nuclear counterstaining, the cardiomyocytes were incubated with 4′,6-diamidino-2-phenylidone (DAPI; Sigma, USA). The presence of troponin I (cTnI) in the cytoplasm of cardiomyocytes could be visualized by green fluorescence, and the DAPI stained nuclei could be visualized in blue fluorescence. Cellular impurities cells did not appear in green fluorescence, only nuclei blue stained. Immunofluorescence images were obtained under a fluorescence microscope (magnification × 100, Olympus, Japan). Five different fields were counted to calculate the purity of cardiomyocytes.

### Evaluation of lentivirus transfection efficiency

Lentivirus carrying fluorescent fragments (GeneChem, China) was prepared separately, and grouped according to the multiplicity of infection (MOI) of 1, 10, and 50 (*n* = 3 per group). The lentivirus was then used to transfect cardiomyocytes in vitro. After 72 h, the transfection rate was observed under a fluorescence microscope (magnification, 200×) to evaluate the optimal MOI, which was used as the transfection dose in subsequent experiments.

### Grouping and cardiomyocyte transfection

After culture for 72 h, cardiomyocytes were divided into six groups (*n* = 3 per group): H_2_O_2_ group, negative control group (NC group), H_2_O_2_ + miR-486 up group, H_2_O_2_ + miR-486 up control group (H_2_O_2_ + UC group), miR-486 down group and miR-486 down control group (DC group). Polybrene (5 μg/ml) was added to each group. For lentiviral vectors (GeneChem, China), the H_2_O_2_ + miR-486 up group and miR-486 down group were respectively transfected with miR-486 precursors (mimics) and reverse sequences (inhibitors), while the corresponding control groups were transfected with control lentivirus vectors with invalid sequences. The H_2_O_2_ group and NC group were treated with equal amounts of phosphate buffered saline (PBS) instead of lentivirus. Cardiomyocytes from each group were used for subsequent experiments after transfection for 72 h.

### Caspase8 inhibits experimental grouping and transfection

After being cultured for 72 h, cardiomyocytes were divided into four groups (*n* = 3 per group): miR-486 down + caspase8 down group (double down group), miR-486 down group, caspase-8 down group and miR-486 control + caspsase8 control group (double control group). Polybrene (5 μg/mL) was added to each group. Then, for lentiviral vectors (GeneChem, China), the double-down group and miR-486 down group were transfected with miR-486 reverse sequences while the remaining two groups were transfected with control lentiviral vectors with invalid sequences. At 1 h before transfection, 10 μmol/L of the caspase-8 inhibitor Z-IETD-FMK was added to the double-down group and caspase-8 down group, and the miR-486 down group and double-control group received the same amount of PBS instead. Cardiomyocytes from each group were then used for subsequent experiments after transfection for 72 h.

### Cardiomyocyte apoptosis modeling

For cardiomyocyte apoptosis, 100, 200 and 400 μmol/L of 30% H_2_O_2_ were separately added to the cardiomyocyte medium to induce apoptosis, and the samples were collected 6 h later and apoptotic indices were detected using flow cytometry to determine the optimal concentration for apoptosis induction. After determining 30% H_2_O_2_ as the optimal concentration, the cardiomyocyte apoptosis model was constructed with the H_2_O_2_ group, H_2_O_2_ + miR-486 up group and H_2_O_2_ + UC group. Meanwhile, the NC group, miR-486 down group and DC group received equal volume of PBS instead of H_2_O_2_.

### Flow cytometry

Flow cytometry was used to detect the apoptosis rate using an Annexin V-APC staining kit (Ebioscience, China). Cardiomyocytes were digested using trypsinogen (Gibco, USA) and collected, centrifuged at 102×g for 3 min, washed with 4 °C D-Hanks (pH = 7.2-7.4), resuspended in 400 μL 1 × binding buffer, stained with 10 μL Annexin V-APC and incubated in the dark at room temperature for 10-15 min, following which the cells were detected within 1 h using Guava easyCyte HT (Millipore, USA).

### TdT-mediated dUTP Nick-end labeling (TUNEL) staining

In vitro cardiomyocytes were prepared in cell climbing slices, and stained using In Situ Cell Death Detection Kit TMR Red (Roche, USA). Three random high-power fields (magnification100×) were photographed using a fluorescence microscope. TUNEL-positive signals were located in the nuclei, where the nuclei of apoptotic cells presented in red fluorescence, and the nuclei of DAPI stained cells were indicated by blue fluorescence. Thus, the cardiomyocyte apoptosis index = number of apoptotic cells/number of DAPI stained cells × 100%.

### Quantitative reverse transcription-polymerase chain reaction (RT-PCR) analysis

Total RNA was extracted using TRIzol reagent (Invitrogen, USA) according to the kit instructions, and quantified using NanoDrop (Thermo Fisher Scientific, USA). miR-486 was reverse transcribed using MicroRNA First Strand cDNA Synthesis (Poly A Tailing, ShengGong, China) and mRNA was reverse transcribed using PrimeScript RT Reagent Kit (Takara RR047A, Japan). The samples were then subjected to PCR with a SYBR Premix Ex TaqII kit (Takara RR820A, Japan), according to the manufacturer’s instructions. RT-PCR was performed using an ABI StepOne system (Applied BioSystems, USA) to detect miR-486, P53, Bbc3 and BCL-2, whose levels were relatively quantified using the 2^–△△^CT method. The internal references for miR-486 and mRNA were U6 and β-actin, respectively. The primer sequences were as follows: miR-486 5′-ATCCTGTACTGAGCTGCCC-3′ (forward), matching sequence provided in MicroRNA First Strand cDNA Synthesis kit (reverse) (Poly A Tailing, ShengGong, China); P53 5′-TCCTCCCCAACATCTTATCC-3′ (forward), 5′-GCACAAACACGAACCTCAAA-3′ (reverse); Bbc3 5′-ACTGCCAGCCTTGCTTGTC-3′ (forward), 5′-AGTCCTTCAGCCCTCCCTTC-3′ (reverse); BCL-2 5′-GACTGAGTACCTGAACCGGCATC-3′ (forward), 5′-CTAGACAGCGTCTTCAGAGACA-3′ (reverse); β-actin 5′-GGAGATTACTGCCCTGGCTCCTA-3′ (forward), 5′-GACTCATCGTACTCCTGCTTGCTG-3′ (reverse); U6 5′-GCTTCGGCAGCACATATACTAAAAT-3′ (forward), 5′-CGCTTCACGAATTTGCGTGTCAT-3′ (reverse).

### Western blot analysis

After the cells were lysed, protein was extracted using a total protein extraction kit (KangChenKC-415, China), and the protein concentration was determined using a BCA Protein Quantification Kit (KangChenKC-430, China). Total protein samples were loaded at 50 μg/well on to sodium dodecyl sulfate (SDS)-polyacrylamide gel for electrophoresis, and then transferred to polyvinylidene fluoride membranes (Millipore, USA), and incubated in 5% BSA solution at room temperature for 1 h. Thereafter, the membranes were treated with the following primary antibodies: anti-P53, anti-Bbc3, anti-BCL2, anti-caspase-8, anti-caspase-3 (all from CST, USA; 1: 1000 dilution), and GAPDH (KangChen, China, 1:10,000), which was used as the internal reference. The membranes were incubated with the antibodies at 4 °C overnight, washed with tris-buffered saline tween (TBST), treated with the horseradish peroxidase-labeled secondary antibodies (KangChen, China; 1:5000 dilution), and incubated at room temperature for 1 h. The membranes were then developed using enhanced chemiluminescence assay and X-ray film exposure (TianNeng, China). The gray-values of the bands were analyzed using ImageJ software.

### Statistical analysis

Statistical analysis was performed using SPSS 23.0 software (IBM Corporation, Chicago, USA). Continuous variables were expressed as mean ± standard deviation ($\overline{x}$ ± s). Comparisons between the two groups or among multiple groups were performed using analysis of variance (ANOVA), followed by post-hoc tests. Cardiac function data was analyzed using two-way ANOVA and post-hoc tests. *P* < 0.05 was considered statistically significant.

## Results

### Identification of cardiomyocytes purity

**Fig. 1:**
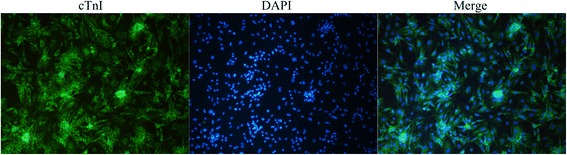
Identification of cardiomyocytes purity. Immunofluorescence and DAPI staining were used to distinguish between cardiomyocytes and other cells. CTnI (*green*) and nucleus (*blue*) were stained in cardiomyocytes, and only the nuclei (*blue*) were stained in other cells. (magnification, 100×)

Immunofluorescence staining was used to identify the purity of cultured cardiomyocytes in vitro (Fig. 1). The purity of cardiomyocytes was found to be 98.67 ± 1.33%.

### Lentiviral transfection of in vitro cardiomyocytes

**Fig. 2:**
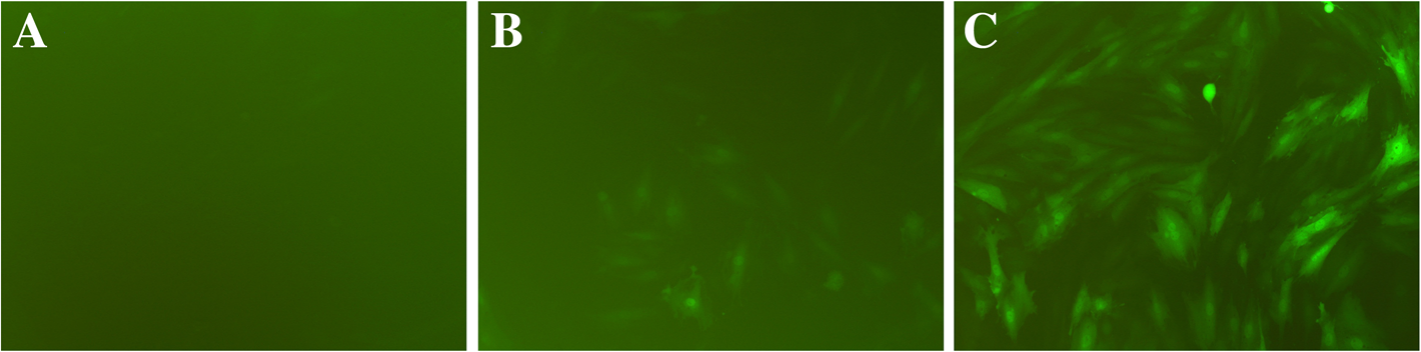
Lentiviral transfection of in vitro cardiomyocytes. **a** MOI = 1, **b** MOI = 10, **c** MOI = 50. Transfection efficiency: **a**: 3.21 ± 0.19%, **b**: 16.72 ± 2.03%, **c**: 89.32 ± 8.35%

In order to determine the optimal conditions for lentiviral transfection in cardiomyocytes in vitro, cardiomyocytes were transfected with lentivirus carrying fluorescence fragments with doses at MOIs of 1, 10, and 50 (*n* = 3 per group). After 72 h, we observed the transfection rates were using a fluorescence microscope, and the rates for these three MOIs were 3.21 ± 0.19%, 16.72 ± 2.03% and 89.32 ± 8.35%, respectively. Considering this, the target cells reached a transfection efficiency of 80% or more as the optimal infection condition, and the required MOI was 50 (Fig. 2).

### H_2_O_2_-induced in vitro cardiomyocyte apoptosis model

**Fig. 3:**
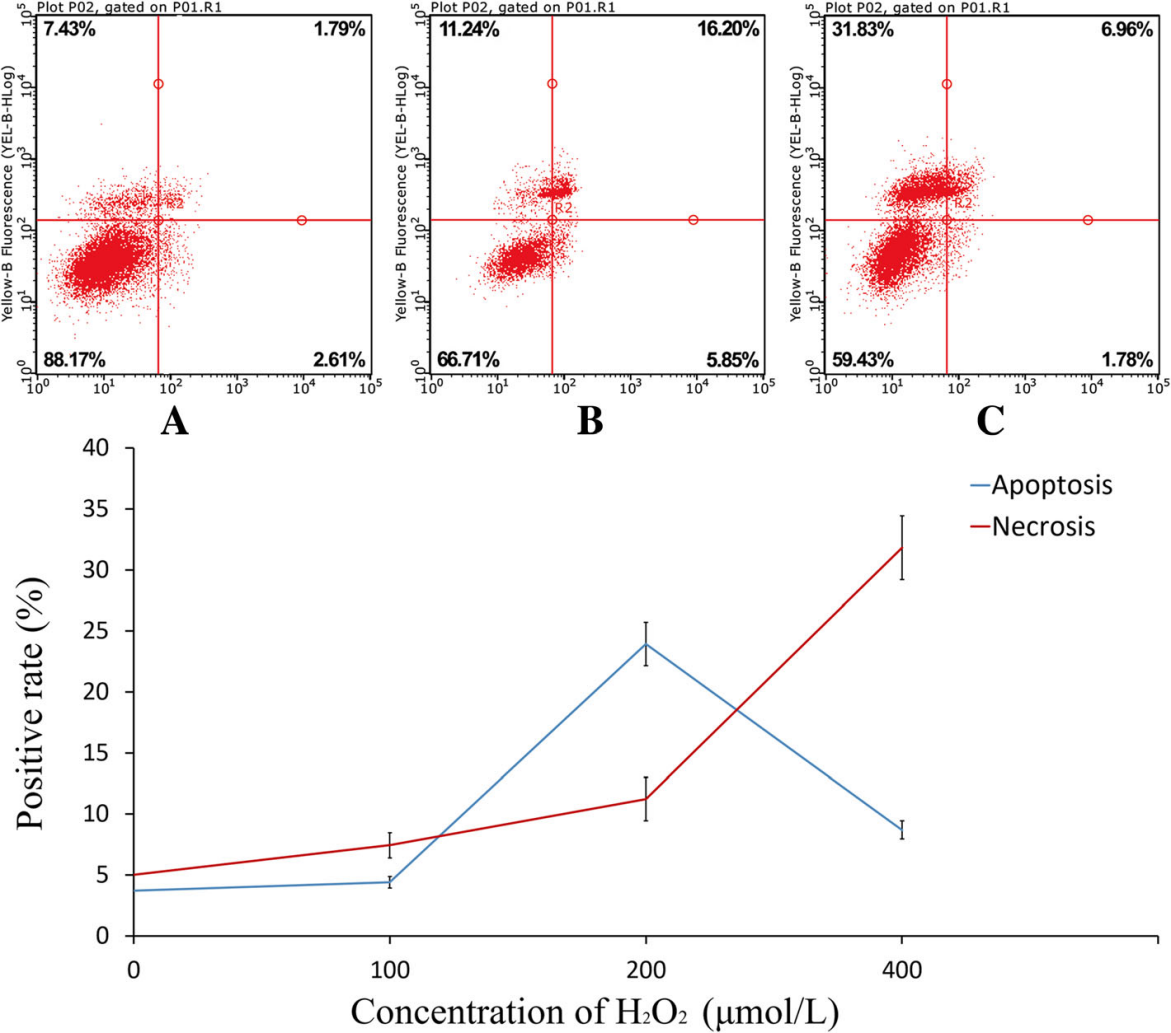
H_2_O_2_-induced in vitro cardiomyocyte apoptosis. Cardiomyocytes were treated with 100 μmol/L H_2_O_2_ (**a**), 200 μmol/L H_2_O_2_ (**b**), 400 μmol/L H_2_O_2_ (**c**). Data were expressed as mean ± standard deviation

H_2_O_2_ is considered as an apoptosis inducer of mature cardiomyocytes. In this experiment, cardiomyocyte apoptosis was induced using different concentrations of H_2_O_2_, and the apoptosis was detected using flow cytometry after 6 h of treatment. The results indicated that cardiomyocyte apoptosis peaked at 23.95 ± 1.78% when 200 μmol/L H_2_O_2_ was used, and cell necrosis was lower at 11.24 ± 1.78%. Thus, 200 μmol/L H_2_O_2_ was considered as an optimal apoptosis inducer (Fig. 3).

### Determination of cardiomyocyte apoptosis

#### Flow cytometry

**Fig. 4:**
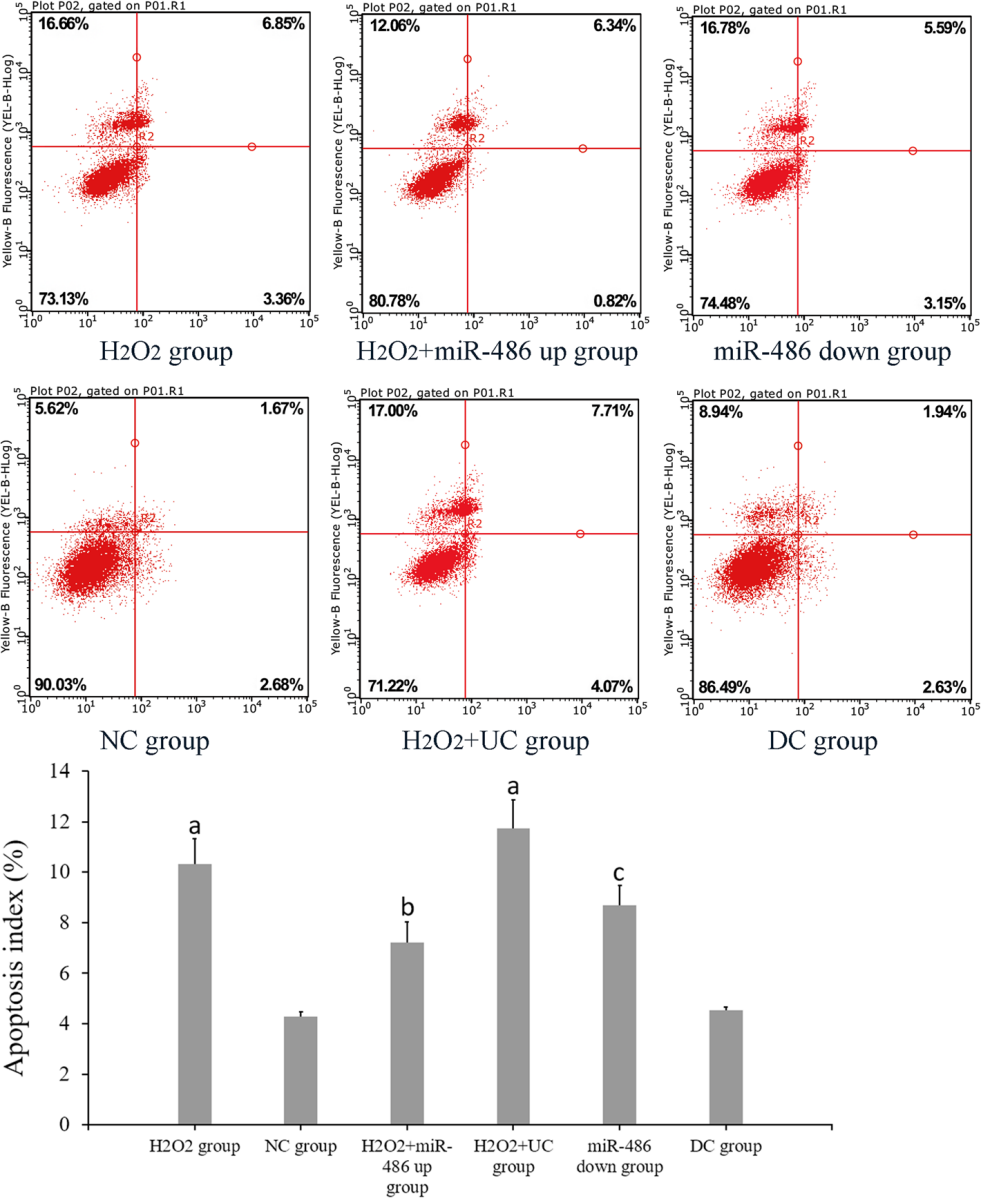
Flow cytometry of apoptosis. Compared with NC group, ^a^ *P* < 0.05. H_2_O_2_ + miR-486 up group compared with H_2_O_2_ + UC group, ^b^ *P* < 0.05. miR-486 down group was compared with DC group, ^c^ *P* < 0.05. NC group: negative control group, H_2_O_2_ + UC group: H_2_O_2_ + miR-486 up control group, DC group: miR-486 down control group

The cardiomyocyte apoptosis rates of the H_2_O_2_ group, NC group, H_2_O_2_ + miR-486 up group, H_2_O_2_ + UC group, miR-486 down group and DC group were 10.32 ± 1.02%, 4.28 ± 0.19%, 7.72 ± 0.82%, 11.73 ± 1.14%, 8.69 ± 0.79% and 4.53 ± 0.14%, respectively (Fig. 4). The cardiomyocyte apoptosis rate was significantly higher in the H_2_O_2_ group and the H_2_O_2_ + UC group than in the NC group (*P* < 0.05), while it was significantly lower in the miR-486 up group than in H_2_O_2_ + UC group (*P* < 0.05), was significantly increased in the miR-486 down group than that in the DC group (*P* < 0.05).

#### TUNEL staining

**Fig. 5:**
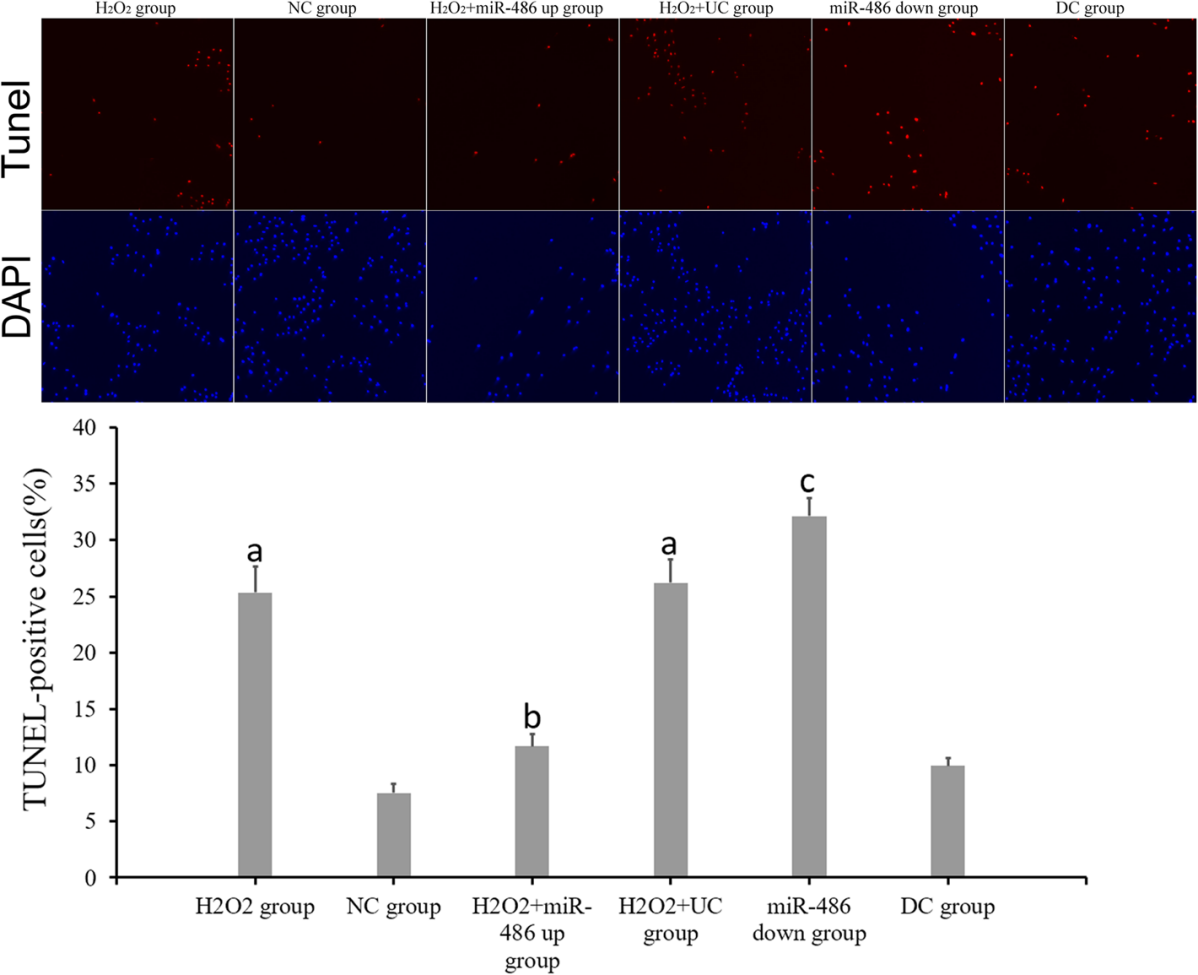
TUNEL staining. Red fluorescence referred to TUNEL-positive cells, DAPI (*blue*) referred to nuclear counterstaining (magnification 100×). ^a^p < 0.05 compared with NC group. ^b^p < 0.05: compared with H_2_O_2_ + UC group, cardiomyocyte apoptosis index was *lower* in the H_2_O_2_ + miR-486 up group. ^c^p < 0.05: compared with DC group, cardiomyocyte apoptosis index was increased in the miR-486 down group

The cardiomyocyte apoptosis index was evaluated using TUNEL staining (Fig. 5). The cardiomyocyte apoptosis indices of the H_2_O_2_ group, NC group, H_2_O_2_ + miR-486 up group, H_2_O_2_ + UC group, miR-486 down group and DC groups were 25.33 ± 2.34%, 7.53 ± 0.81%, 11.71 ± 1.05%, 26.21 ± 2.09%, 32.17 ± 1.61% and 9.96 ± 0.69%, respectively. Thus, the cardiomyocyte apoptosis index was significantly lower in the H_2_O_2_ + miR-486 up group than in the H_2_O_2_ + UC group (*P* < 0.05), while it was significantly increased in the miR-486 down group than in the DC group (*P* < 0.05, Fig. 4).

### RT-PCR results of miR-486, p53, Bbc3 and BCL-2 expressions (Figs. 6, 7, 8 and 9)

1. Compared with the NC group, the p53 and Bbc3 expression levels were significantly increased in the H_2_O_2_ group and H_2_O_2_ + UC group (*P* < 0.05), while the miR-486 and BCL-2 expression levels were significantly decreased (*P* < 0.05).
2. Compared with the H_2_O_2_ + UC group, the p53 and Bbc3 expression levels were significantly lower in the H_2_O_2_ + miR-486 up group (*P* < 0.05), while the miR-486 and BCL-2 expression levels were significantly increased in this group (*P* < 0.05).
3. Compared with the DC group, the p53 and Bbc3 expression levels were significantly increased in the miR-486 down group (*P* < 0.05), while the miR-486 and BCL-2 expression levels were significantly decreased (*P* < 0.05).

**Fig. 6:**
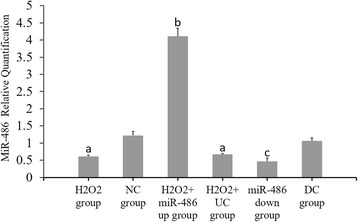
Fluorescence-based quantitative PCR results of miR-486 expression. Compared with NC group, ^a^ *P* < 0.05. H_2_O_2_ + miR-486 up group compared with H_2_O_2_ + UC group, ^b^P < 0.05. miR-486 down group compared with DC group, ^c^ *P* < 0.05

**Fig. 7:**
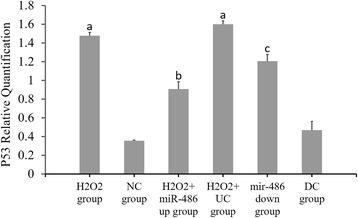
Fluorescence-based quantitative PCR results of p53 mRNA expression. Compared with NC group, ^a^ *P* < 0.05. H_2_O_2_ + miR-486 up group compared with H_2_O_2_ + UC group, ^b^P < 0.05. miR-486 down group compared with DC group, ^c^ *P* < 0.05

**Fig. 8:**
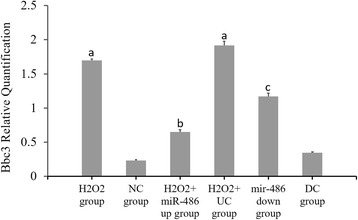
Fluorescence-based quantitative PCR results of Bbc3 mRNA expression. Compared with NC group, ^a^ *P* < 0.05. H_2_O_2_ + miR-486 *up* group was compared with H_2_O_2_ + UC group, ^b^P < 0.05. miR-486 *down* group compared with DC group, ^c^ *P* < 0.05

**Fig. 9:**
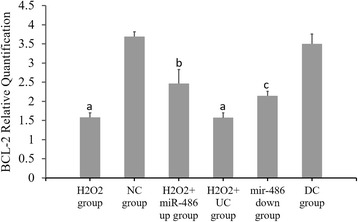
Fluorescence-based quantitative PCR results of BCL-2 mRNA expression. Compared with NC group, ^a^ *P* < 0.05. H_2_O_2_ + miR-486 up group compared with H_2_O_2_ + UC group, ^b^P < 0.05. miR-486 down group compared with DC group, ^c^ *P* < 0.05

### Western blot analysis (Figs. 10, 11, 12 and 13)

1. Compared with the NC group, the relative expression levels of p53, Bbc3 and cleaved caspase-3 proteins were significantly increased in the cardiomyocytes of the H_2_O_2_ group and H_2_O_2_ + UC group (*P* < 0.05), while the relative expression levels of BCL-2 protein was significantly decreased (*P* < 0.05).
2. Compared with the H_2_O_2_ + UC group, relative expression levels of p53, Bbc3 and cleaved caspase-3 proteins of the H_2_O_2_ + miR-486 up group were significantly lower (*P* < 0.05), while that of BCL-2 protein was significantly increased (*P* < 0.05).
3. Compared with the DC group, relative expressions of p53, Bbc3 and cleaved caspase-3 proteins of the miR-486 down group were significantly increased (*P* < 0.05), while BCL-2 protein expression showed a significant decline in the miR-486 down group (*P* < 0.05).

**Fig. 10:**
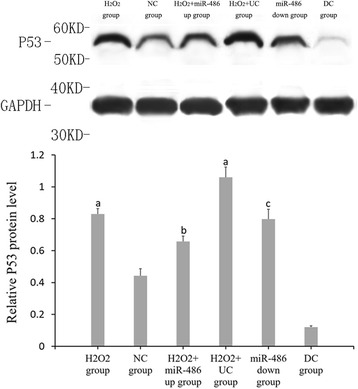
Western blot results of relative expression of p53 protein. Compared with NC group, ^a^ *P* < 0.05. H_2_O_2_ + miR-486 up group compared with H_2_O_2_ + UC group,^b^P < 0.05. miR-486 down group compared with DC group,^c^ *P* < 0.05

**Fig. 11:**
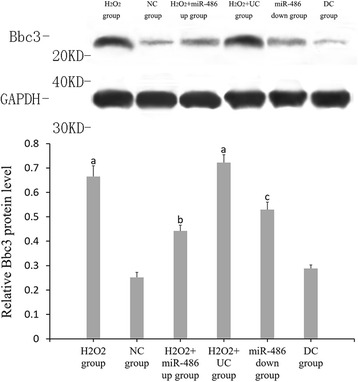
Western blot results of relative expression of Bbc3 protein. Compared with NC group, ^a^ *P* < 0.05. H_2_O_2_ + miR-486 up group compared with H_2_O_2_ + UC group,^b^P < 0.05. miR-486 down group compared with DC group,^c^ *P* < 0.05

**Fig. 12:**
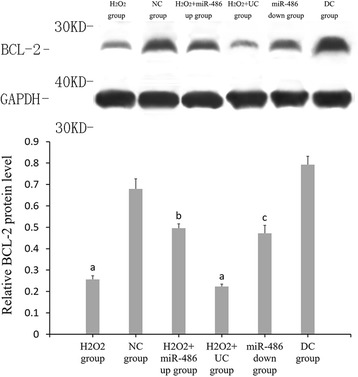
Western blot results of relative expression of BCL-2 protein. Compared with NC group, ^a^ *P* < 0.05. H_2_O_2_ + miR-486 up group compared with H_2_O_2_ + UC group,^b^P < 0.05. miR-486 down group compared with DC group,^c^ *P* < 0.05

**Fig. 13:**
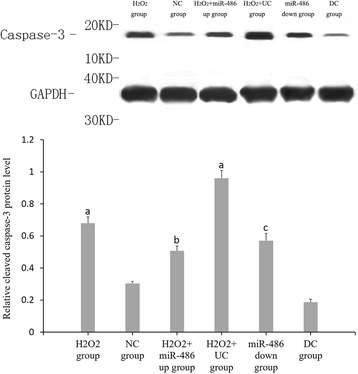
Western blot results of relative expression of cleaved caspase-3 protein. Compared with NC group, ^a^ *P* < 0.05. H_2_O_2_ + miR-486 up group compared with H_2_O_2_ + UC group,^b^P < 0.05. miR-486 down group compared with DC group,^c^ *P* < 0.05

### Correlation analyses between miR-486, p53, Bbc3, BCL-2, cleaved caspase-3 and cardiomyocyte apoptosis

MiR-486 expression showed a significant negative correlation with p53 (*r* = −0.681, *P* < 0.001), Bbc3 (*r* = −0.761, *P* < 0.001) and cleaved caspase-3 (*r* = −0.639, *P* = 0.001); but had significant positive correlation with BCL-2 (*r* = 0.805, *P* < 0.001), and significant negative correlation with cardiomyocyte apoptosis (*r* = −0.805, *P* < 0.001). P53 expression had a significantly positive correlation with Bbc3 (*r* = 0.769, *P* < 0.001) and cleaved caspase-3 (*r* = 0.752, *P* = 0.035), and a negative correlation with BCL-2 (*r* = −0.773, *P* < 0.001). Meanwhile, Bbc3 expression was positively correlated with cleaved caspase-3 (*r* = 0.682, *P* = 0.005) and was negatively correlated with BCL-2 (*r* = −0.732, *P* = 0.028).

### Caspase-8 inhibition experiment

**Fig. 14:**
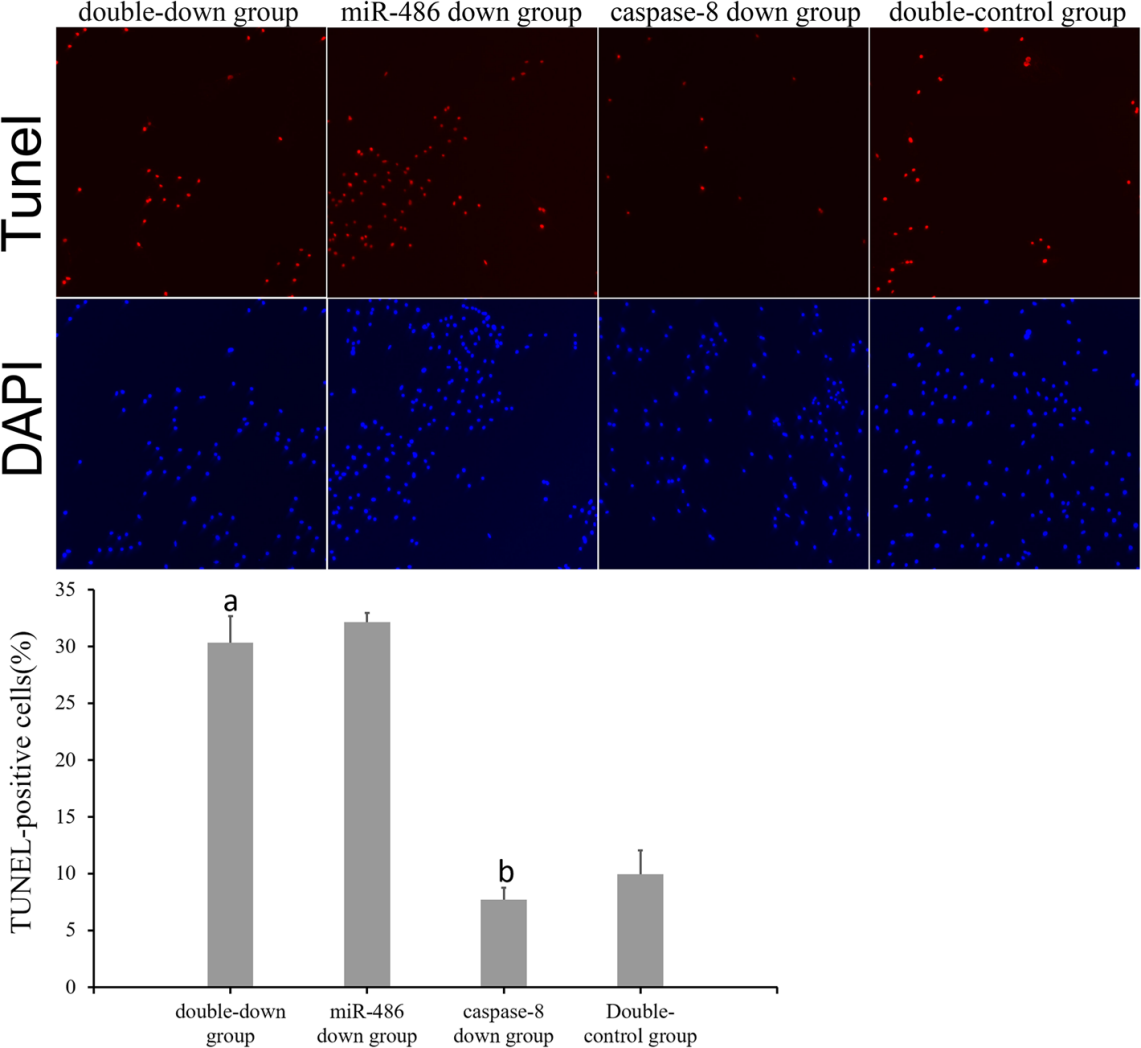
TUNEL staining. TUNEL-positive cells are indicated by red fluorescence, and nuclei counterstained with DAPI are stained blue (magnification 100×). *Double-down* group compared with the caspase-8 down group, ^a^ *p* < 0.05. Caspase-8 down group compared with the double-control group, ^b^ *p* < 0.05. The cardiomyocyte apoptosis index demonstrated no significant difference between the double-down group and the miR-486 down group (*P* > 0.05)

**Fig. 15:**
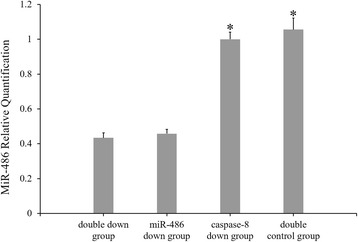
Fluorescence-based quantitative PCR results of miR-486 expression. Compared with the double-control group, ^*^ *P* < 0.05

**Fig. 16:**
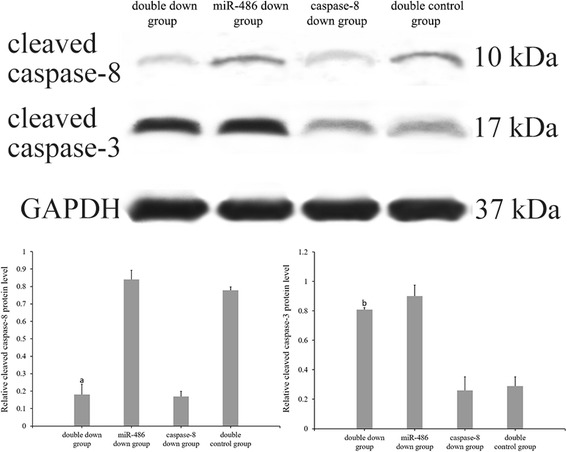
Western blot analysis of cleaved caspase-8 and cleaved caspase-3 protein levels. Compared with the miR-486 down group, ^a^ *P* < 0.05. Compared with the caspase-8 down group, ^b^p < 0.05

1. TUNEL staining (Fig. 14): Cardiomyocyte apoptosis indices of the double-down group, miR-486 down group, caspase-8 down group and double-control group were 30.33 ± 2.34%, 32.17 ± 1.61%, 7.71 ± 1.05% and 9.96 ± 0.69%, respectively. The cardiomyocyte apoptosis index did not significantly differ between the double-down group and the miR-486 down group (*P* > 0.05), but it was significantly higher in the double-down group than in the caspase-8 down group (*P* < 0.05). The apoptosis index was significantly lower in the caspase-8 down group than in the double-control group (*P* < 0.05).
2. RT-PCR result of miR-486 (Fig. 15): The miR-486 expression levels were significantly lower in the miR-486 down group and double-down group than in the double-control group (*P* < 0.05, in both cases), while it did not significantly differ in the caspase-8 down group.
3. Western blot analysis of cleaved caspase-8 and cleaved caspase-3 (Fig. 16): The relative expression level of cleaved caspase-8 protein was significantly lower in the double-down group than in the miR-486 down group (*P* < 0.05), while that of cleaved caspase-3 protein did not significantly differ between the two groups (*P* > 0.05). The relative expression level of cleaved caspase-8 protein did not significantly differ between the double-down group and caspase-8 down group (*P* > 0.05), while cleaved caspase-3 protein level significantly increased in the double-down group relative to the caspase-8 down group (*P* < 0.05). Additionally, the relative cleaved caspase-3 protein expression slightly decreased in the caspase-8 down group relative to the double-control group, although this difference was not significant (*P* > 0.05).

## Discussion

Cardiomyocyte apoptosis is involved in many physiological and pathophysiological processes, and is considered as the cytological basis for the occurrence and evolution of a variety of cardiovascular diseases [17–19]. In vitro experiments have indicated that the activation mechanism of apoptosis is complex, the mitochondrial pathway, the death signal receptor pathway and various signaling pathways are involved in the occurrence and development of cardiomyocyte apoptosis [20], which leads to reduced cardiac contraction function, decreased pump function, disorder of electrical activities, severe heart failure or even death [21]. In this study, we used H_2_O_2_ to induce apoptosis in primary cardiomyocytes in vitro to detect the correlation between the p53-mediated BCL-2 associated mitochondrial apoptotic pathway and apoptosis in cardiomyocytes, and to study the regulatory effect of miR-486 on this pathway.

P53 is one of the major cytokines that initiates apoptosis in cardiomyocytes [22], and can regulate Bbc3, tp53-induced glycolysis and apoptosis-regulator(TIGAR), and other factors to further activate the death signal receptor pathway. Bax/BCL2, NF receptor, Fas protein and other pathways are also known to regulate apoptosis [9, 23]. Bbc3 is a member of the BH3-only subfamily, and plays an important role in apoptosis as a target gene for p53 [24]. Bbc3 interacts with Bcl-2 and Bax, and changes the permeability of the mitochondrial membrane permeability [25, 26]. Budhram et al. found that p53 overexpression in cardiomyocytes up-regulated Bbc3 expression, which in turn aggravated cardiomyocyte apoptosis in a hypoxia-reoxygenation model [5]. In this study, we found that H2O2 treatment increased cardiomyocytes apoptosis, upregulated the expression levels of p53 and Bbc3 and significantly downregulated BCL-2 expression, and increased the cleaved caspase-3 protein level. Furthermore, p53 was positively correlated with cardiomyocyte apoptosis and with Bbc3 and cleave caspase-3 expression levels, but was negatively correlated with BCL-2. This suggests that p53 activation significantly upregulates Bbc3 expression during cardiomyocyte apoptosis and, in turn, affects the expression levels of downstream BCL-2 and caspase-3.

MiR-486 has been proved to intervene with apoptosis by modulating PTEN, PIM-1 and other effectors [27, 28]. Meanwhile, Hall et al. found that miR-486 was involved in the regulation of p53-induced DNA damage [29]. Peng et al. found that miR-486 was negatively correlated with p53 expression, and ultimately affected the expression of cleaved caspase-3 [30]. In our preliminary experiment, microarray results revealed that miR-486 expression was significantly down-regulated in cardiomyocyte apoptosis, and was negatively correlated with p53 level and cardiomyocyte apoptosis rate. In the present study, to further verify the relationship between miR-486 and P53/Bbc3/BCL-2 associated with the mitochondrial apoptotic pathway in cardiomyocyte apoptosis, we established a miR-486 silenced model and H_2_O_2_-induced cardiomyocyte apoptosis model with miR-486 overexpression. Our results demonstrated that miR-486 expression was significantly decreased followed by H_2_O_2_-induced cardiomyocyte apoptosis, indicating that miR-486 expression was inhibited in cardiomyocyte apoptosis. We also found that miR-486 overexpression could significantly suppress the expression fo p53, Bbc3, cleaved caspase-3, and up-regulate BCL-2 expression in the H_2_O_2_ + miR-486 up group but not in the H_2_O_2_ + UC group, thereby reducing the incidence of cardiomyocyte apoptosis. It is clear that miR-486 upregulation may contribute to the activation of BCL-2-related mitochondrial apoptotic pathways, thereby exhibiting anti-apoptotic function in cardiomyocyte apoptosis. Meanwhile, miR-486 silencing up-regulated p53, Bbc3 and cleaved caspase-3 expression levels, and downregulated BCL-2 expression, which then increased cardiomyocyte apoptosis rate. This result suggested that miR-486 decline may have relieved p53 inhibition and the downstream apoptotic pathway, resulting in loss of the protective effect on cardiomyocytes. All these results were line with our hypothesis that miR-486 is involved in the pathological process of cardiomyocyte apoptosis by regulating their response to the p53-dependent BCL-2 associated mitochondrial apoptotic pathway.

The report showed that the death receptor pathway was also involved in cardiomyocyte apoptosis, and caspase-8 was one of the important factors independent of mitochondrial apoptosis that could activate caspase-3 to induce apoptosis [20]. To avoid the effect of the death receptor pathway on this study, we performed a caspase-8 inhibition experiment. To study this, miR-486 was downregulated and cleaved caspase-8 expression was also inhibited, and the cleaved caspase-3 expression and apoptosis levels were analyzed. When cleaved caspase-8 was inhibited, cleaved caspase-3 expression decreased slightly and cardiomyocyte apoptosis also decreased, indicating that the death receptor pathway was indeed involved in cardiomyocytes apoptosis. In addition, cleaved caspase-3 expression and apoptosis were both found to increase when miR-486 was downregulated, and this change was not affected by caspase-8 downregulation. This finding suggested that miR-486 still had a significant effect on caspase-3 activation and cardiomyocyte apoptosis even after excluding the interference of the death receptor pathway. We speculated that the death receptor pathway may not play a major role in miR-486 regulation of cardiomyocyte apoptosis.

The limitation of this study is in the usage of in vitro cardiomyocytes, which they do not fully represent the integrated heart, and thus cannot be used to simulate the complex physiological mechanisms of the entire organ. Also, the single cardiomyocyte apoptosis model cannot represent the entire apoptotic stimulus environment, and the biological characteristics of cardiomyocytes from neonatal rats and adult rats have some differences. Thus, certain differences may exist in the pathophysiological changes between the experimental and actual disease conditions.

## Conclusions

In summary, we demonstrated that miR-486 was significantly down-regulated in apoptosis of neonatal rats’ cardiomyocytes. MiR-486could influence the process of cardiomyocyte apoptosis by regulating P53-mediated BCL-2 associated mitochondrial apoptotic pathway. MiR-486 overexpression in cardiomyocytes can effectively reduce the activation of p53-mediated BCL-2 apoptotic pathway, thus protecting the cardiomyocytes, and conversely, reduced miR-486 expression would increase cardiomyocyte apoptosis. Therefore, the possibility of exploiting miR-486 as a novel drug target for some kinds of heart diseases should be further explored in future studies.

## Abbreviations

ANOVA: Analysis of variance
DAPI: 4′,6-diamidino-2-phenylindole
DC: Down control
FBS: Fetal bovine serum
miR: microRNA
MOI: Multiplicity of infection
NC: Negative control
PBS: Phosphate buffered saline
PTEN: Phosphate and tension homology deleted on chromosome ten
RT-PCR: Reverse Transcription-Polymerase Chain Reaction
TBST: Tris-buffered saline tween
TUNEL: TdT-mediated dUTP Nick-End labeling
UC: Up control
